# A54 DIETARY COMPONENTS ARE ASSOCIATED WITH FECAL CALPROTECTIN IN ULCERATIVE COLITIS

**DOI:** 10.1093/jcag/gwab049.053

**Published:** 2022-02-21

**Authors:** E Chiu, L Taylor, R Ingram, R Panaccione, S Ghosh, H Ramay, K McCoy, R Reimer, M Raman

**Affiliations:** Medicine, University of Calgary, Calgary, AB, Canada

## Abstract

**Background:**

Ulcerative colitis (UC) is thought to arise from dysregulated immune responses due to intestinal dysbiosis and altered epithelial barrier function. Dietary components may affect the gut microbiome and contribute to either inflammation or its resolution. The relationship between diet and disease activity in UC warrants further investigation.

**Aims:**

This prospective cohort study explored the relationship between dietary components, and markers of disease activity: fecal calprotectin (FCP) and partial Mayo score (PMS) in patients with UC.

**Methods:**

40 participants were recruited from University of Calgary IBD clinics. Study staff obtained two 24-hour diet recalls using the validated automated self administered (ASA)-24 and captured PMS at baseline (T1) and follow-up at week 12 (T2). FCP samples were collected at T1 and T2. Diet variables included adjusted macro/micronutrients (n=44), food groups (n=36) and the validated Canadian healthy eating index-2009 (CHEI) where higher scores reflect healthier intake. CHEI captures intake of dark green and orange foods (DGO) and moderation scoring (MOD) of saturated fats (SF), sodium and added sugars. Higher CHEI scores result from increased intake of DGO and lower intake of SF, sodium and added sugars (higher MOD score). Associations with outcome variables were examined at T1 and T2 individually and across both timepoints (BT). Mixed effect logistic regression models identified relationships between dietary variables, FCP and PMS. Models were adjusted for age, sex, BMI, medications, probiotics, and for repeated measures in both timepoint analyses.

**Results:**

A positive association was identified between FCP as a continuous variable and SF (T1:Coef=0.22, p_adj=0.02) and a negative association identified between FCP with citrus/melon/berries (BT:Coef=-1.01, p_adj =0.04), total sugars (BT:Coef=-0.06, p_adj=0.025) and HEI (BT:Coef=-0.13, p_adj =0.06 and T1 coef=-0.18, p_adj =7.0 e-5). FCP increased as SF (-0.30,p_adj=0.01), DGO (-0.60, p_adj=0.02), and MOD (-0.21, p_adj=0.02) scores decreased. The presence of inflammation (as a binary variable, FCP >250) was negatively associated with higher fiber intake (BT: Odds Ratio (OR)= 0.016, CI(0.001,0.40) p_adj=0.08). For PMS as a continuous variable, HEI had a negative association with PMS (T2: -0.05, p_adj=0.06). With PMS as a discrete score (remission=PMS<2) there was no significant association with any diet components.

**Conclusions:**

This study suggests that a healthier diet, both in overall pattern and specific dietary components, was associated with lower FCP and PMS. Our findings related to SF, citrus/melons/berries, and DGO parallel the IOIBD dietary guidelines. Future research should explore through controlled intervention studies whether modifying dietary patterns and components independently reduces disease activity.

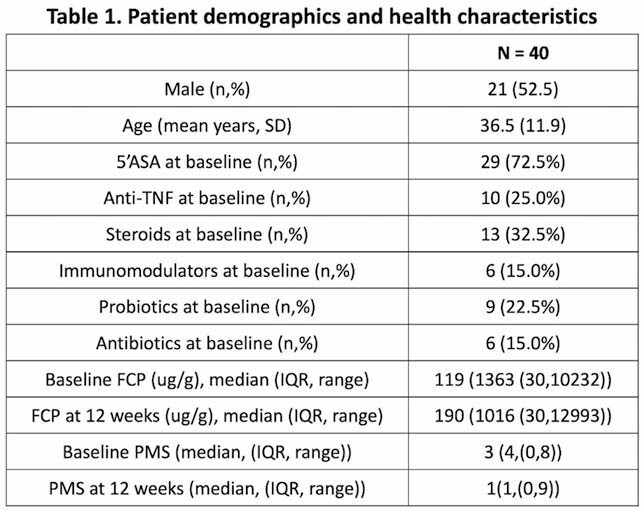

**Funding Agencies:**

Crohn’s and Colitis Foundation

